# A Pilot Study Exploring Gender Differences in Residents’ Strategies for Establishing Mentoring Relationships

**DOI:** 10.3885/meo.2008.res00263

**Published:** 2008-05-25

**Authors:** Megan C. McNamara, Melissa A. McNeil, Judy Chang

**Affiliations:** * Division of General Internal Medicine, University of Pittsburgh, Pennsylvania. Montefiore University Hospital, Suite 933W, 200 Lothrop Street, Pittsburgh, PA, 15213; † Department of Obstetrics, Gynecology, and Reproductive Sciences, University of Pittsburgh, Magee Women's Hospital, 300 Halket Street, Pittsburgh, PA, 15213

**Keywords:** Residents, mentoring, gender

## Abstract

**Background:**

Mentoring is important throughout a physician's career and has been noted to be particularly important during residency training. Other studies suggest that women may experience difficulty in finding mentors.

**Purpose:**

This study explored gender-specific differences in residents’ mentoring experiences.

**Methods:**

The authors conducted two focus groups at the University of Pittsburgh in July, 2004. One group was composed of 12 female residents; the other was composed of nine male residents. Discussions were audiotaped and transcribed. Two investigators coded the transcripts and identified emerging themes.

**Results:**

Residents of both genders cited multiple barriers to mentoring. Men′s strategies for finding mentors were more numerous than women′s and included identifying mentors through research, similar interests, friendship, and networking. Female strategies were limited and included identifying mentors through “word of mouth” and work experiences. Women described more passive approaches for finding a mentor than men.

**Conclusions:**

Female residents may lack strategies and initiatives for finding mentors. Residency programs should create opportunities for residents to develop mentoring relationships, with special attention paid to gender differences.

Mentoring is important throughout a physician's career, but may be especially critical at times of professional transitions, such as residency.^1^,^2^ Mentoring has been linked to residents’ career choices, interest in academic medicine, and personal growth.^3^–^6^ Residents perceive mentoring as beneficial, and most program directors favor mentoring.^6^,^7^ The Accreditation Council for Graduate Medical Education (ACGME) has highlighted the importance of mentoring in residents’ professional development.^8^,^9^

Women, in particular, are thought to benefit substantially from mentoring. Women with mentors report greater job satisfaction, are more likely to be promoted, and spend more time engaged in scholarly activities.^10^,^11^ Conversely, inadequate mentoring can have significant consequences for female trainees.

Female residents may experience problems with stress and confidence, and are less likely to establish a solid career network when compared to their male counterparts.^4^,^12^ Among medical students, women are discouraged from certain specialties because of a paucity of role models, and cite lack of mentorship as a significant barrier in their medical education.^13^,^14^ Interest in academic medicine is more likely to decrease among women, as compared to men, during residency; mentoring may be instrumental in preventing this decline. 4

Formal mentoring programs, which assign a faculty mentor to a resident, ensure that all residents are mentored; however, these relationships may be less effective than those formed spontaneously.^3^,^15^ Most residents prefer “self-initiated” mentoring relationships, but finding a suitable mentor takes time, which is in short supply for busy residents.^16^,^17^ Female residents, in particular, may face additional obstacles in establishing mentoring relationships. In a recent systematic review of physician mentoring, women experienced more difficulty in finding mentors than their male counterparts.^18^ Among 4,721 surveyed Obstetrician-Gynecology residents, white female residents reported the fewest mentoring relationships. 19 Additionally, female residents interested in General Internal Medicine were less likely than male residents to identify a potential mentor.^20^

Gender differences in initiating mentoring relationships may result from a number of factors. A lack of women in leadership positions may result in fewer potential mentors for women.^21^ Social interactions which promote spontaneous mentoring, such as participating in sports, may be less accessible to women.^22^ Differences in social behaviors between men and women may also be important; women, who are traditionally assigned a more passive role, may be hesitant ask for mentorship.^22^ However, it is unclear if any of these factors are relevant to residents seeking mentors.

Mentoring relationships are nuanced interactions which are ideally explored using qualitative research methods. Few qualitative studies of residents’ mentoring relationships exist in the literature; a recent systematic review specifically excluded qualitative studies. The purpose of our study was to understand gender differences in residents’ experiences with mentoring. We asked female and male residents to describe the barriers they encounter in finding a mentor and the strategies they use to overcome these barriers. Using residents’ own words, we can gain a better understanding of the most effective methods for ensuring that all residents have access to mentoring.

## Methods

**Study Design -** This study used a grounded theory approach to explore residents’ experiences with mentoring during residency. We elicited residents’ personal accounts about their experiences with mentoring during residency training using focus group interviews. Our study used focus groups because we felt that residents would be more comfortable talking about this topic in a group setting, and that the exchange of shared experiences would facilitate thoughtful and rich discussion. The study was reviewed and approved by the Institutional Review Board at the University of Pittsburgh prior to the start of the study.

**Sampling -** We collaborated with the program directors of six core training programs at the University of Pittsburgh Medical Center to recruit residents in Internal Medicine, Pediatrics, General Surgery, Family Medicine, Psychiatry, and Obstetrics and Gynecology for two focus groups, one with male residents and one with female residents. We chose to recruit from these specialties because they each have accredited programs which train a large number of residents every year.

Our goal was to recruit a total of 24 residents (12 female, 12 male). We used a combination of stratified random and quota sampling strategies to achieve equal representation by gender across the specialties and levels of training. Program directors for each specialty provided the names of 366 current trainees for the 2004–2005 academic year; these names were further stratified according to training level. From these names, the PI selected 54 men and 54 women to invite for participation in the study; estimating that 20% of invitations would be accepted. Names were randomly selected from within each stratum. Potential subjects were invited to participate through a personalized email invitation; this invitation described the purpose of the study, the dates and times of the focus groups, and compensation. Residents responded to this invitation by emailing the PI directly. The PI then phoned each of the respondents, described the study in further detail, and confirmed the resident's interest. A second reminder phone call was made just prior to each focus group. We conducted the focus groups after regular work hours at a central site on the University of Pittsburgh Medical Center campus. Residents were compensated with $25 for their study participation.

**Data collection -** Each participant completed a brief anonymous questionnaire at the beginning of the focus group session. This questionnaire collected data on age, race, training level, future career plans, and current involvement in a mentoring relationship. A trained moderator conducted each focus group, and an observer took notes. Each focus group was audiotaped and lasted 1.5 hours. We asked each group three questions to stimulate discussion regarding their experiences with mentoring during residency and their strategies for finding a mentor. These questions were reviewed by local experts in qualitative analysis at the University of Pittsburgh, and were thought to be sufficiently open-ended and general to stimulate discussion. These questions were: “What do you think of when you hear the word ‘mentor’?”; “What have your mentoring experiences during residency been like?”; “How do you find a mentor who is helpful to you?”

**Data analysis-** Each session was transcribed verbatim and both the moderator and observer reviewed each transcript to make sure it matched their recollection of the discussion. In our analysis, we used a variety of methods to corroborate our interpretation of the data; these steps helped to ensure consistency and prevent bias. Two coders independently analyzed each transcript and assigned codes to individual words, phrases, and sentences. They then met and compared their coding. There were no differences in interpretation of the data; differences in the application of specific coding terms to a particular phrase or section were discussed and resolved by consensus. The list of codes was iterative; a final coding scheme was developed from the list. This final coding scheme was applied to the transcripts and thematic trends were identified. Additional methods to corroborate our findings included the following: 1) review of analysis among a larger study group; 2) review of analysis by focus group participants; 3) review of analysis by a former residency program director, a current residency program director, a local expert in resident education, and a local expert in mentoring; and 4) peer review in formal work-in progress sessions and invited presentations. These reviewers found the themes we presented to be plausible based on their own experiences.

## Results

**Participants –** Among the 108 residents who were contacted, 30 residents (18 female, 12 male) volunteered for the focus groups. The first 12 female residents who responded to the email invitation were recruited for participation in the study. Three of the 12 male residents who volunteered did not come to the scheduled focus group and thus did not participate in the study; thus, the total sample included 21 residents (12 women and 9 men). Specialties represented included Internal Medicine, Pediatrics, General Surgery, Family Medicine, and Obstetrics/Gynecology; years of training ranged from post-graduate year 1 to post-graduate year 7. Nine of the female residents and five of the male residents reported that their residency had a mentoring program. More detailed information regarding the composition of each group and the characteristics of our study participants is provided in the Table 1. Among women who participated in a residency with a mentoring program, Seven reported that they had been mentored, one indicated “minimal” mentorship, and one reported that she had not been mentored. One male resident indicated that, despite training in a residency with a mentoring program, he had not been mentored.

**Table 1.** Characteristics of Study Participants

Characteristic	Female Residents (N = 12)	Male Residents (N = 9)
Age		
25–29 years	9	7
30–34 years	3	2
Year of training		
Post Graduate Year 1	2	2
Post Graduate Year 2	5	5
Post Graduate Year 3	4	
Post Graduate Year 4		2
Post Graduate Year 7	1	
Specialty		
Internal Medicine	3	5
General Surgery	3	3
Pediatrics	3	1
Obstetrics/Gynecology	2	
Family Medicine	1	
Residency has mentoring program		
Yes	9	5
No	2	3
Not sure	1	1
Has been mentored during residency		
Yes	10	4
No	2	4

**What the residents said -** Residents had participated in mentoring relationships by being assigned a mentor through their residency program, or by finding a mentor on their own. Residents of both genders expressed similar views regarding assigned mentoring and barriers to establishing mentoring relationships. However, male and female residents articulated different approaches and strategies for finding a mentor. In the following section, individual quotes are italicized; when known, the specialty of the respondent is listed in parentheses.

**Residents’ perceptions about assigned mentoring -** Residents did not view assigned mentors as “true” mentors. Assigned mentors were perceived as advisors, and these relationships were typically transient and impersonal: “*But I think very often that first person everyone gets assigned to in residency is any advisor who may change.” (male, Pediatrics); “They…have a list of 30 other people they're advising.” {male, Internal Medicine}*.

Trainees had few expectations for assigned relationships, and seldom anticipated that assigned faculty would become genuine mentors. At best, assigned mentoring relationships served as a springboard for identifying other, more fruitful relationships: “*Sometimes maybe assigned things can work as a launching block.” (male, Pediatrics).*

At worst, such relationships could be considered an obstacle, delaying the resident from developing a relationship that really worked. Residents agreed that the best mentoring relationship were “found” but noted that this process was challenging; they welcomed assistance in identifying faculty mentors: “*I think it's better if somebody gives you guidance as to how to go about finding a mentor.” (male, Internal Medicine).*

**Barriers to establishing mentoring relationships -** Residents perceived multiple barriers to establishing mentoring relationships. Busy call schedules and multiple clinical responsibilities often took priority for residents, and identifying a mentor necessarily assumed less importance. Residents had to be dedicated to the process of finding a mentor, because no “official” time was allocated for this process: “*How can you find (mentors) except by bum luck or true motivation in finding the spare time and by the grace of God ‘having the day off’ or the half hour for lunch.” (male, Pediatrics).*

A perceived lack of interested and accessible faculty mentors represented another barrier for residents trying to establish mentoring relationships. Faculty members’ busy schedules and multiple responsibilities often rendered potential mentors unapproachable: “*I think particularly at the academic level, there are a lot of people who are really great at what they do, but don't have a lot of time for being a mentor and so to have a mentor who… serves as a great role model and is doing a lot of really impressive things is great, but not if you can't ever talk to them or be around them.”(female, Internal Medicine).* Even faculty mentors who had committed to the relationship made residents feel that they were being squeezed in. One resident described a meeting with her mentor: “*Once was in the cafeteria for ten minutes because we couldn't connect… And it was kind of like ‘So how are things going?’ It was like, how can I tell you my whole intern year, my experience from emotional to physical? I talk fast, but not that fast.”(female, Pediatrics).*

Trainees felt that residency programs did little to foster effective mentoring relationships. Residents commented that they were “on their own” in seeking a mentor, and that programs failed to cultivate an atmosphere of mentoring: “*I went to (a university) where it seemed like they lived and breathed mentoring…And then I cam here and it was the total opposite. It didn't seem as if anyone was interested.” (female, Surgery).* Residents expressed a sense of abandonment and isolation, and noted they were receiving little assistance from faculty: “*So now I'm kind of like doing it on my own. And I figured it out, but it's kind of like…the process was done without any kind of help.” (male, Internal Medicine).*

**Residents’ strategies for finding a mentor -** Male and female residents employed distinctly different strategies for finding a mentor. Men's strategies were more numerous than women's, and included identifying mentors through:

1. **Research** (*“So I would be able to spend time doing research and probably cultivate the relationship based on that.” [male, Internal Medicine]*);
2. **Similar interests** (*“If you're in some sports…you can have a mentor do the same activities at the same time.” [male, Internal Medicine]*);
3. **Fiendship** (*“You become friends with them.” [male, Surgery]*);
4. networking (*“And sometimes the role of the mentor is just teaming you up with someone who is a better mentor for you.”[male, Pediatrics]*);
5. **Looking outside the work environment** (*“And finding the perfect mentor sometimes will only happen outside of this system.”[male, Internal Medicine]*).

Female strategies were limited and included identifying mentors through:

1. **Word of mouth** (*“One of the upper level residents will recommend somebody to you.”[female, Pediatrics]*);
2. **Work experiences** (*“Just you're put together in a situation on a team or in a location.”[female, Obstetrics/Gynecology]*).

Men reported strategies indicating they took initiative in developing mentoring relationships and actively sought out mentors: “*If you strike up a conversation and happen to strike an interest, follow it up. Don't let it go by. Seize the moment.”(male, Pediatrics*). If a mentoring relationship was not successful, male residents took steps to identify a new mentor: “*They set us up with a faculty advisor, but he was an anesthesiologist. I'm like, that's great, but how about some surgeon friend. He gave me three names and I went and met this guy.” (male, Surgery).* Male residents surrounded themselves with a “pool of eligible mentors,” and then chose among those one who would be most helpful for their career focus. Similarly, they worked to maintain the relationship once it was established by combining mentoring with other activities: “*So it's good if you can do two things at the same time, you know. That can help deal with the restraint of time.”(male, Internal Medicine).*

In contrast, women described more passive approaches to finding a mentor. Women worried about being a “bother” to faculty mentors, and seemed concerned that faculty may not be interested in, or willing, to help them. Most were reluctant to initiate contact with mentors, preferring instead that the mentor show interest first: “*They will offer sometimes too, which is really nice because then you know they're approachable because they approached you.”(female, Internal Medicine).* A few of the female residents were willing to approach a mentor if they identified one with similar career goals: “*So I kind of got to know one of the gastroenterologists and he's kind of been mentoring me a little bit along the way.” (female, Internal Medicine).*

For many female residents, mentoring relationships that developed usually resulted from chance circumstances, such as being paired with an interested faculty member on a clinical rotation. In those situations, female residents were more comfortable letting the relationship evolve, rather than formally asking for mentoring: “*I have never gone to someone and said, ‘Do you want to be my mentor?” (female, Pediatrics).* Even when women recognized the need for career advice from a mentor, many were hesitant to approach one: “*It sort of felt like it was up to me to really find someone that was more in tune to what I want to do.* [interviewer: And have you done that?] *Not really yet.”* (*female,* *Pediatrics)*. Female residents expected mentors to continue to “prove” their interest once the relationship had developed. Mentors should be the ones to arrange meetings, initiate communication, and follow-up with the resident: “*I think it's important for a mentor to be the one to say ‘let's get together’.” (female, Obstetrics-Gynecology).*

## Discussion

In this qualitative pilot study, we found that residents face a variety of challenges in forming mentoring relationships. Although these barriers were perceived equally by male and female residents, there were gender-specific differences in residents’ methods for overcoming these barriers. As compared to male residents, we found that female residents reported fewer strategies for finding a mentor and were more passive in their approach to forming mentoring relationships.

Studies of female residents have demonstrated that they are less likely to identify mentors, and receive mentoring, compared to their male colleagues. 18,19,20,23 Our study may provide some explanation for this observation. Protégés are typically responsible for seeking a mentor; most authorities recommend adopting a strategic approach in this process.^17^,^21^,^24^ Searching for someone with similar interests; engaging in outside activities; and looking outside one's own institution, have all been advocated as useful ways for fostering mentoring relationships.^17^,^21^, ^25^ In this study, male residents reported using these strategies, as well as a variety of others, to generate a “pool” of potential mentors. In contrast, women enumerated few methods for identifying mentors and did not articulate a cohesive strategy for doing so.

Women in our study were reluctant to seek out faculty, and assumed that mentors would approach them. This may in part be due to socialized behaviors, in which women are taught to assume more traditionally passive roles in initiating relationships.^22^,^26^ Researchers have speculated that mentoring challenges faced by women in health care may arise from a clash between social roles and a male-dominated hierarchal medical culture.^27^ Given the lack of female mentors in leadership positions, women may hesitate to initiate a relationship with a male mentor.^22^ Since individuals who actively seek mentoring are more likely to receive it, women's lack of initiative may limit their success in obtaining good mentors. Additionally, male residents were effective at strategically assembling an informal network of mentor and advocates to guide their careers; such networks are often considered essential for success.^27^ Women's inability to create this network may lead to isolation and missed opportunities.

Many residency programs have recognized the challenges trainees face in finding mentors, and have developed assigned mentoring programs.^15^ Several of the residents in our study had assigned mentors, and most agreed that these relationships were transient and impersonal. Similar findings have been documented previously. Among psychiatry residents, assigned mentoring was thought ineffectual because it lacked a “personal element”; in other studies, residents favored mentoring relationships that were formed by free choice, rather than by assignment.^3^,^16^ These results suggest that merely assigning faculty mentors to female residents is in an inadequate strategy for ensuring that they experience beneficial mentoring relationships.

Our study has limitations that deserve mention. This descriptive study used qualitative methods and purposive sampling, and its results cannot be generalized to all residents. Due to funding constraints, we were also limited to two focus groups. We also recognize that thematic saturation may not have been achieved with two focus groups. Although we corroborated all emerging themes through member-checking and expert review, a larger number of focus groups might provide additional information. Despite including residents from a variety of specialties and training levels, our study design and size limited any comparisons that we could make based on these characteristics. Finally, we did not explore faculty mentors’ views about resident mentoring. Further study is needed to investigate any perceived gender differences in mentoring from the perspectives of faculty mentors.

The information obtained in this study has important implications. Training programs should acknowledge the importance of resident mentoring and create an environment that prioritizes it for both residents and faculty. Special attention should be paid to the needs of female residents, who may need guidance to successfully find or use mentors. Women residents should be educated about the importance of mentorship, given various strategies to initiate contact with potential mentors, and encouraged to actively seek and maintain their mentoring relationships. Potential mentors should recognize that they may need to approach women and offer mentorship. Additionally, programs may need to consider creative approaches to ensuring mentoring of residents. One possibility is to combine an assigned mentor/selected mentor approach by providing an assigned mentor for the first year with the expectation that this mentor assist the resident in identifying and establishing a relationship with a mentor more appropriate to the resident's interests and career goals. This mixed approach may be potentially useful in assisting female residents find good mentors. Further research is needed to obtain a richer understanding of gender differences in residents’ mentoring relationships; knowledge gained from such studies could inform the development of residency mentoring programs specifically tailored to meet all residents’ needs.
